# Measurement Efficiency of a Teacher Rating Scale to Screen for Students at Risk for Social, Emotional, and Behavioral Problems

**DOI:** 10.3390/jintelligence11030057

**Published:** 2023-03-19

**Authors:** Gino Casale, Moritz Herzog, Robert J. Volpe

**Affiliations:** 1 School of Education, Institute for Educational Research, University of Wuppertal, Gaußstraße 20, 42119 Wuppertal, Germany; 2 Department of Applied Psychology, Northeastern University, Boston, MA 02115, USA

**Keywords:** universal screening, item response theory, behavior problems, school-based assessment

## Abstract

Teacher rating scales are broadly used for psycho-educational assessment in schools. In particular, they play an important role in screening students for social, emotional, and behavioral problems. In order to optimize the efficiency of these measures, it is important to minimize the number of items comprising them while maintaining sound psychometric characteristics. This study examines the measurement efficiency of a teacher rating scale for student social, emotional, and behavioral risk. The goal was to shorten an existing behavior screening tool. A total of 139 classroom teachers and 2566 students from Grades 1–6 (M_age_ = 8.96 years, SD = 1.61) participated in the study. In sum, 35 items assessing internalizing and externalizing behavior problems were analyzed applying the item response theory (generalized partial credit model). The results show that social, emotional, and behavioral risks can be captured with a total of 12 items. This reduction of almost 66% of the initial item pool would take teachers about 90 s to fill out for one student. Thus, the rating scale can be used by teachers in an efficient yet psychometrically sound manner.

## 1. Introduction

### 1.1. Social, Emotional, and Behavioral Competencies in Children and Adolescents

The social, emotional, and behavioral development of children and adolescents plays a central role in primary education. Social and emotional competence is a broad and multidimensional construct for which many different operationalizations and models exist (Berg et al. 2019). At its core, social–emotional competence refers to interpersonal and intrapersonal skills in the emotional (e.g., emotion knowledge and emotion regulation), social (e.g., social problem solving, processing social cues), and cognitive (e.g., executive functions) domains (Berg et al. 2019). 

Among other things, these skills are associated with academic performance, school success, and the development of psychosocial disorders (Aviles et al. 2006; Domitrovich et al. 2017). A large proportion of all school-age children and adolescents shows significant impairments in social, emotional, and behavioral development. Depending on the definition used and the informants involved in generating estimates, approximately 12–18% of children and adolescents with emotional and behavioral disorders can be identified internationally (Kovess-Masfety et al. 2016; Polanczyk et al. 2015). Among these, internalizing disorders, such as anxiety, occur more frequently than externalizing difficulties, such as attention-deficit/hyperactivity disorder (ADHD; Kovess-Masfety et al. 2016).

A variety of school-based interventions can promote social, emotional, and behavioral competencies in students. In three meta-analyses (Durlak et al. 2011; Korpershoek et al. 2016; Sklad et al. 2012), building prosocial behavior, reducing behavior problems, and increasing academic achievement was effective with a small effect size; increasing social– emotional skills was effective with a medium effect size.

However, there is often a significant gap between the initial presence of a student’s social, emotional, and behavior problems and the provision of school-based interventions (Daniels et al. 2014). It is estimated that only about 20–30% of all children and adolescents with problems in social, emotional and behavioral development receive systematic support in terms of prevention or intervention (Langer et al. 2015). Although these numbers differ between countries, this “service gap” (Forness et al. 2012, p. 3) is widespread and concerning. One reason for this “underservice” is that many students with problems in their social, emotional, and behavioral development remain unidentified and their problems are not recognized until they already correspond to symptoms of a clinical disorder (e.g., Breitenstein et al. 2009). This problem precludes the application of early support services that have been shown to be effective in preventing the escalation of developmental trajectories (e.g., Durlak et al. 2011). Alternatively, early identification of the aforementioned problems can promote prevention and counteract the development of mental disorders (e.g., Costello 2016).

### 1.2. Early Identification of Social, Emotional, and Behavioral Risk in Students

Both the externalizing and internalizing behaviors of students are significant indicators of the social and emotional competence of children and adolescents. Externalizing behavior problems have a significant impact on positive social interactions in the classroom and disrupt learning and teaching processes (Lane et al. 2014). Therefore, these behavioral problems are often better and more accurately identified by teachers than internalizing behavioral problems, which are often overlooked (e.g., Dwyer et al. 2006; Hartman et al. 2017). For this reason, among others, it is important to provide teachers with tools that can be used for the early identification of students’ externalizing and internalizing behavioral problems (Splett et al. 2019).

Many different approaches exist for the assessment of social, emotional, and behavioral characteristics in children and adolescents, e.g., behavioral observations, test batteries, or more innovative approaches such as situational judgement tests or forced choice assessments (Halle and Darling-Churchill 2016). These methods usually show acceptable to good psychometric characteristics, but are often very time-consuming in regard with preparation, implementation, and evaluation, which is incompatible with everyday school routines. As such, they may not be suitable for the universal screening of at-risk students.

For an initial assessment of whether students are exhibiting problems in the social, emotional, and behavioral domains, universal screening methods for student behavioral problems have proven effective within a decision-making process in an evidence-based assessment (Volpe and Briesch 2018). Universal behavior screening tools “are conducted with all students in a classroom […] to identify those at-risk of behavioral difficulties or emotional and behavioral disorders (EBD) who could potentially benefit from specific instruction or intervention” (Glover and Albers 2007, p. 118). Eklund et al. (2009) showed that the use of universal screening procedures identified more than twice as many at-risk students as other psychoeducational assessment practices. Ideally, a consequent result of this early detection of at-risk students is the provision of interventions at the first sign of these problems (Volpe et al. 2010).

In general, universal behavioral screenings work by having teachers complete ratings for each student. The results can be used to make decisions regarding student risk for developing severe social–emotional behavioral problems. However, several studies show that far fewer than half of all schools and teachers systematically screen their students for social, emotional, and behavioral risks (Bruhn et al. 2014; Dineen et al. 2022; Glover and Albers 2007; Wood and Ellis 2022). This still strongly underutilized use of universal screenings can be attributed in part to the overly broad scope of many standardized screening instruments, which tend to discourage teachers from using them (Burns and Rapee 2019; Volpe et al. 2018). One important predictor of the implementation of universal screening procedures is the teachers’ attitudes towards screening (Moore et al. 2022). Teachers’ attitudes towards universal screening are mainly affected by the required resources for implementation, especially the time teachers need for completion (Briesch et al. 2017; Kauffman 1999). Therefore, one critical feature of universal screening tools should be that they are highly time-efficient, but still beneficial for practical use in schools.

An established procedure for the time-efficient screening of social, emotional, and behavioral risks in children in school is multiple-gating (Walker et al. 2014). The basic idea behind multiple-gating procedures is to progressively narrow down the pool of potential at-risk students by using increasingly rigorous methods at each successive gate. This approach is also promoted as best practice in screening in school contexts (Whitcomb and Merrell 2013), and has been shown to be superior to using a procedure involving a single measure (Kilgus et al. 2018). Efficiency is gained in this approach if time-efficient measures are used in earlier gates to rule out typically developing students with more time-intensive methods used for the remaining students. Multiple-gating procedures often have three stages (see Stiffler and Dever 2015): first, the teacher nominates students who the teacher subjectively perceives as exhibiting social, emotional, and behavioral problems. A comparatively short broadband rating scale is then completed for the students who advance to the second gate. A third gate could either consist of a systematic direct observation of a small pool of students or a more comprehensive rating scale. 

### 1.3. Measurement Efficiency of Universal Behavior Screenings

Following Glover and Albers (2007) and Volpe and Briesch (2018), universal behavior screening procedures should meet three essential requirements: (1) Appropriateness for the intended use (i.e., alignment with constructs of interests and theoretical and empirical support); (2) Technical adequacy of the tool (i.e., psychometric properties); and (3) Usability of the tool (i.e., cost–benefit ratio, acceptability, and utility of outcomes). With regard to school-based universal screening, the appropriateness for the intended use is given if the tool provides timely and useful information regarding the levels of risk for all students (Daniels et al. 2014). In the school context, the constructs of interest are not clinically relevant symptom scales, but rather behavioral scales that capture problems in social, emotional, and behavioral dimensions (see Volpe et al. 2018). Technical adequacy indicates that the screener demonstrates acceptable reliability, validity, and accuracy in the early identification of at-risk children (i.e., classification accuracy). Usability implies that: (a) The tool is feasible and acceptable to stakeholders; and (b) The results of the screener guide the selection of interventions (Glover and Albers 2007).

This third category of usability also includes the aspect of measurement efficiency (e.g., Anthony et al. 2016). By measurement efficiency we mean that the preparation, implementation, and interpretation of the measurement instrument are carried out with the least possible time effort while obtaining the best possible psychometric information (Anthony et al. 2016). With reference to behavior rating scales, this means that the number of items to be completed is minimized, but these items are still representative for the underlying latent constructs, and thus, the results can be used meaningfully to identify at-risk students (Glover and Albers 2007). If these psychometric requirements are met, the results of the screening can be used to distinguish between students with and without social, emotional, and behavioral risk.

In order to make the best selection of items for these purposes from a test theory perspective, it is important to obtain the most comprehensive and accurate information possible. Item response theory (IRT; e.g., Wilson 2004) is suitable for this purpose. In the context of IRT, the difficulty of the items (as manifest variables) is examined in relation to the actual trait expression of the subjects (as latent variables). For universal screening, this means the social, emotional, and behavioral problems of a student (latent trait) and the specific items (manifest traits) correspond accordingly (Anthony et al. 2016). IRT analyses could be used to map how well the items differentiate between different levels of competence (in this case, between students with and without risk). This approach also allows an analysis of which items are particularly salient and meaningful in classifying between at-risk and non-at-risk students, so that the results can be used for optimal item selection and reduction (Hambleton 2000).

### 1.4. The Current Study

The current study represents a re-analysis of data published by Volpe et al. (2020) with results from using the integrated teacher reporting form (ITRF; Volpe and Fabiano 2013) to improve measurement efficiency for social, emotional, and behavioral risk. The instrument is considered a well-established universal screening for primary school students that includes 35 items related to internalizing and externalizing classroom behaviors, such as depressive behavior (AD), socially withdrawn behavior (SW), oppositional/disruptive behavior (OPD) and academic productivity behavioral problems (APP). The aim of the present study is to increase the measurement efficiency of the scale by reducing the number of items to a minimum level required to accurately discriminate between at-risk and non-at-risk students. More specifically, we were interested in retaining the items of the full ITRF that:

(a) Discriminate best between children with low and high levels of behavioral problems; and

(b) Are sensitive to students with above-average behavioral problems, but not necessarily very high problems. As students with very high levels of behavioral problems are the most likely to be identified by teachers (even without an assessment tool), early universal screening should detect even mild-to-moderate behavioral problems (Kendziora 2004). 

While meeting the above-mentioned criteria, we seek to delineate a shortened version of the ITRF, which is comparable to the full-length version in regard to its ability to discriminate students with and without significant behavioral problems.

## 2. Materials and Methods

### 2.1. Participants and Setting

A total of 10 inclusive primary schools, 2 inclusive secondary schools, and 3 special schools from one school district in the federal state North Rhine Westphalia (NRW; Western Germany) participated in the study. In sum, 139 classroom teachers completed the questionnaires for 2566 students (48.2% female). The mean age of the teachers was 43.00 years (SD = 9.28), with a mean teaching experience of 15.84 years (SD = 8.96). The mean age of the student sample was 8.96 years (SD = 1.61), with a range from 6 to 15 years. The majority of the students was from Grades 1 to 4 (91.2%), 8.8% were from Grades 5 and 6. Regarding gender, 90.4% of the teachers were female. Information about the study and the data collection processes were provided by a member of the research team at a school principal meeting and additional personal communication (e.g., phone calls and mailing) before the data collection started. All schools received a packet containing ITRF forms, and an additional form to record the sociodemographic characteristics of students. Each individual classroom teacher completed both forms for all the students in the classroom and sent them back to the investigators.

### 2.2. Instrument—The Integrated Teacher Report Form (ITRF) 

The ITRF was initially developed to assess the externalizing behavioral problems of primary school students in the classroom (Volpe and Fabiano 2013). The English-language ITRF was translated into German and adapted and validated for use in both a long and a short version (Casale et al. 2018; Volpe et al. 2018). In addition, the instrument was expanded and validated with items referring to internalizing classroom behaviors (Volpe et al. 2020). This version assesses student externalizing and internalizing classroom behaviors that indicate a social, emotional, and behavioral risk (Volpe et al. 2020). It consists of 35 items (see Appendix A) measuring academic productivity problems (8 items), oppositional/disruptive behavior (8 items), anxious/depressive behavior (11 items), and social withdrawal (8 items). The ITRF is part of the Integrated Screening and Intervention System (Volpe and Fabiano 2013), which incorporates universal screening, intervention, and behavioral progress monitoring. Numerous studies support its factorial validity, internal consistency, retest reliability (Daniels et al. 2014; Volpe et al. 2018, 2020), construct validity (Casale et al. 2019), and cross-cultural equivalence (Casale et al. 2018). In particular, those studies examined how the ITRF relates to other established behavioral screening measures. However, those studies only included the externalizing scales of the ITRF. Daniels et al. (2014) tested convergent and discriminant validity and used a symptom-based behavioral assessment for teachers in addition to the ITRF (brief problem monitor; Achenbach et al. 2011). High correlations between content-like constructs and low correlations between content-distant constructs underscore the construct validity of the ITRF. For the German-language version, the classification accuracy and predictive validity for identifying a problem of the ITRF was analyzed (Volpe et al. 2018). For this purpose, the Teacher Report Form of the Child Behavior Checklist (TRF-CBCL; Achenbach et al. 2008) was used as the criterion measure. The calculation of receiver operating curves (ROC) and positive as well as negative predictive values (PPV & NPV) indicated a high diagnostic accuracy for all scales of the externalizing ITRF (AUC .85–.94). For all scales, NPVs were substantially higher than PPVs, which is acceptable for a screening procedure because more students are selected for intervention than are actually prevalent psychosocial problems (Volpe et al. 2018). Finally, in another study with the German-language ITRF, convergent and discriminant validity were analyzed using a multitrait–multimethod correlation matrix and a correlated trait–correlated method minus 1 model to separately analyze the influence of the constructs (learning-related/attentive behavioral problems, oppositional/disruptive behavioral problems) and the methods (ITRF, additional assessment procedure) on the resulting scores (Casale et al. 2019). The additional screenings were the strengths and difficulties questionnaire (SDQ; Goodman 1997), the TRF-CBCL, and the *Lehrereinschätzliste für Sozial- und Lernverhalten* (LSL; Petermann and Petermann 2013; teacher assessment schedule for social and learning behavior). The results demonstrate that the theoretically postulated correlations can be mapped to the empirical data, in line with expectations, indicating convergent and discriminant validity. The variance of the ITRF values can be explained to a greater extent by the construct being measured than by method-specific influences, which also supports the construct validity of the ITRF. In addition, Volpe et al. (2018) conducted a systematic comparison of the externalizing ITRF with established German-language screening procedures (SDQ, TRF-CBCL, LSL) in terms of their usability for school-based use. The results demonstrate that except for the ITRF, none of the instruments are fully suitable for use in schools because they are either too symptom-orientated (TRF-CBCL), too comprehensive (TRF-CBCL, LSL), or not systematically linked to school-based interventions (SDQ, TRF-CBCL) (Volpe et al. 2018).

In this study, the participating classroom teachers completed the full-length ITRF for all the students in their classroom in order to precisely identify the problematic classroom behaviors raising most of the concern for the students. The teachers completed the ITRF items on a 4-point Likert scale (0 = behavior is not of concern, 1 = behavior is of slight concern, 2 = behavior is of moderate concern, and 3 = behavior is of strong concern).

### 2.3. Analysis Design

**Figure 1:**
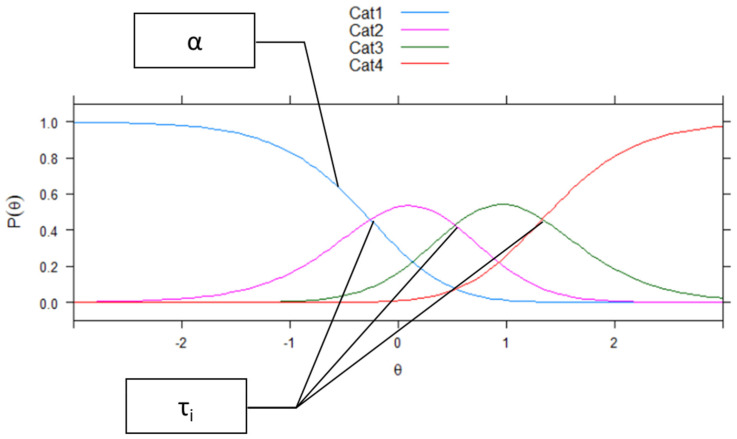
Typical ICC of a polytomous item and item parameters α (discrimination) and τ_i_ (threshold location). θ refers to the latent trait. P(θ) refers to the probability of the answer categories. The different colors of the curves refer to the different answer categories.

To identify the items of the full ITRF that discriminate well between students with low and with high levels of behavioral problems, and that measure especially slightly above the population mean, we applied item response theory (IRT) models, in particular the generalized partial credit model (GPCM). IRT models measure a latent trait (e.g., behavioral problems) on the same scale as the corresponding items (the theta (θ) continuum). That means that for each item, a location on the theta continuum can be estimated (Parameter β). In terms of questionnaires, this parameter can be interpreted as the likelihood with which raters will rate a higher score at this item (or “agreeability”). Given that IRT models are probabilistic models, the location on the theta continuum is defined as the level of the underlying trait at which the probability of being scored higher increases the most (P(θ)). Given a limited amount of answer options (e.g., on a Likert-type scale), when items are dichotomous (e.g., yes or no), IRT models only report one parameter of “agreeability”; however, when items are polytomous (e.g., never, sometimes, often, and very often), there are several thresholds estimated that indicate the level of the underlying trait at which the most probable answer changes (e.g., from never to sometimes). As these parameters (τ_i_) indicate the borders between the most probable answers, there is one parameter less than for the answer options. The GPCM has the advantage that the steepness in which the probability of being scored higher increases can be differentiated between the items (Parameter α) (Muraki 1997). This parameter indicates how strongly the item discriminates between persons with a high trait and a low trait. The probability of multiple answers (e.g., in a Likert scale) across the theta range can be illustrated in the item characteristic curve (ICC). While dichotomous items only have on curve (e.g., for the category “right”), polytomous items have several curves—one for each answer option. Figure 1 shows a typical ICC for an item with four answer options and also illustrates the item parameters α and τ_i_.

The IRT analyses are structured in two sections. First, the items of the full version of the ITRF were reduced. Based on the parameters of the GPCM, items showing the highest discrimination (values of α) and a comparably low “agreeability” (values of β and τ_i_) were selected for retention. Since IRT models require that the items under investigation measure a unidimensional construct, items of the ITRF were divided into four subscales (AD, SW, OPD, and APP), as indicated by Volpe et al. (2020). The selected items for each subscale were taken as potential shortened versions of the full-length ITRF subscales. Second, the internal and external validity of the new versions was investigated. Internal validity was checked by Crombach’s α. To investigate to what extent the full version and the shortened versions of the ITRF correspond, correlations between the sum scores were calculated.

All analyses were conducted in *R* (R Core Team 2022) using the packages *TAM* (Test Analysis Modules; Robitzsch et al. 2022) and *psych* (Procedures for Psychological, Psychometric, and Personality Research; Revelle 2022).

## 3. Results

In total, four GPCMs were employed, one for each subscale of the ITRF. Two main assumptions have to be fulfilled before applying IRT models to the data. First, the data has to be unidimensional. This means that the items included in the model cover the same construct. Usually, unidimensionality is investigated via factor analysis. Given the factor analysis provided by Volpe et al. (2020), the four subscales of the ITRF are unidimensional and distinct from each other. 

Second, the data have to be locally independent. That means that there are rarely covariations among the items. Typically, Q_3_ statistics between the item pairs of a data set are used to check for local dependency (LD). There are different critical values of the Q_3_ statistic discussed in the literature. However, 0.2 and 0.3 appear to be often used as critical values for LD (Christensen et al. 2017). To test for LD, item pairs were formed within the subscales of the ITRF. Of a total of 139 item pairs, 103 (74%) showed a Q3 statistic below 0.2, 28 item pairs (20%) had a moderate Q_3_ between 0.2 and 0.3, and eight item pairs (6%) had a considerable LD with a Q_3_ statistic above 0.3. 

LD is a common problem in data that were rated by several individuals (Anthony et al. 2016; Wu 2017). LD in such cases is often caused by general tendencies (e.g., trend to the middle) and individual tendencies (e.g., leniency) in rating behavior (Wu 2017). Song (2019) showed that LD compromises the results of a GPCM only to a small degree. As the aim of this study was not to assess individuals’ traits in detail, but to compare item characteristics, GPCMs still appear adequate. 

The main basis for the item reduction in the four subscales of the ITRF was the degree of discrimination (α) and the item location (i.e., the range of the underlying trait where the item measures best; β). Based on the item characteristics, three items from each subscale were selected for the shortened version of the ITRF. Three selection criteria were applied: First, high discrimination between persons with low and high behavioral problems (high parameter α). Second, low item location within the latent trait continuum (low parameter β). Additionally, third, a small theta range in which “no difficulties” was the most probable answer category (low parameter τ_1_). Table 1 comprises the information on discrimination, item location, and theta range for τ_1_. Finally, in terms of content, we examined whether the items that met the aforementioned psychometric criteria also matched the underlying constructs in terms of content and were not too similar in content or redundant.

**Figure 2:**
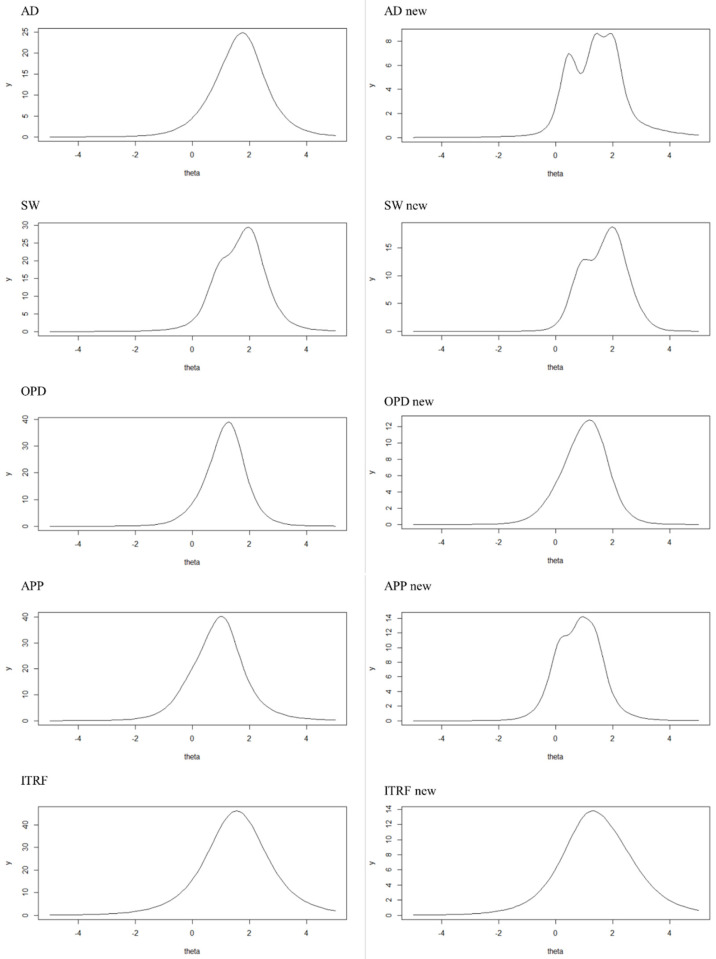
Test information curves for subscales and full questionnaire of the original (**left**) and shortened (**right**) version. Anxious/depressed behavior; SW = social withdrawal; OPD = oppositional/defiant problems; APP = academic productivity problems; ITRF = integrated teacher report form.

To check to what extent the shortened version of the ITRF is more sensitive in the middle theta range, test information curves (TICs) were plotted. TICs display the information an item (collection) provides across the theta range. The shape of a TIC can inform, in which theta range (e.g., little or severe behavioral problems) the focus of test information of an item collection lies. Figure 2 shows the TICs of the subscales and the full scale of the original ITRF and the shortened version. The TICs illustrate that the information focus of the shortened subscales AD, SW, and APP had shifted to the theta range of between 0 and 1 compared to the full versions. In the subscale OPD, the information focus had only slightly shifted to the theta range between 0 and 1. However, as in the subscale OPD, as the items that had the lowest localization on the theta range (parameter β) had already been selected, no further optimization would be possible. Regarding the full ITRF, the test information of the shortened version had slightly shifted to the theta range between 0 and 1. 

To check if the shortened version of the ITRF has the same factor structure as the original version—and thus, if the subscales of the shortened version can be used to assess children’s differential behavioral problems—a confirmatory factor analysis was employed (see Table 2). A model fit of the confirmatory factor analysis was acceptable (CFI = .954, TLI = .937, RMSEA = .075, C.I._RSMEA_ = [.70–.80]) and all factor loadings were significant (*p* < .001). Factor loadings ranged from .65 to .86, and thus, confirmed the four-factor structure of the shortened version of the ITRF. The scale intercorrelations between both externalizing factors (r = .457, CI: .451–.464) and between both internalizing scales (r = .425, CI: .418–.432) were moderate (Table 3). The intercorrelations between the externalizing and internalizing factors were low to moderate (r = .160–.346). The internal consistency of the full scales and the subscales SW, OPD, and APP of the shortened version of the ITRF were good (Cronbach’s α between .85 and .87). Additionally, the internal consistency of the subscale AD was acceptable (Cronbach’s α = .73).

In a final step, the concordance of the full scale and the subscales of the original and the shortened version of the ITRF were investigated. For all subscales and the full scale, the shortened and the original version correlated strongly (*r* > .78). 

## 4. Discussion

The aim of this study was to maximize the measurement efficiency of a teacher rating scale for the school-based assessment of social, emotional, and behavioral risk in students. IRT models were applied in order to analyze the potential to reduce the number of items of a well-established universal screening scale, the ITRF (Volpe and Fabiano 2013). The test information of the shortened version was supposed to be more focused on the theta range between 0 and 1 in order to be more sensitive to children with moderate social, emotional, or behavior problems. Finally, the shortened version had to measure similarly to the original version of the ITRF, including the factor structure. The shortened version proposed in this study meets all these criteria. 

Our analyses indicate that the social, emotional, and behavioral risk of students can be assessed with 12 items only (three items per construct), which is a reduction of almost 66% of the original scale. Speaking in terms of time, and assuming a processing time of the original ITRF of about 5 min per student, the time required to complete the scale for a student can be reduced to about 90 s. For a universal screening of an entire school class of approximately 25 students, this means that the ITRF can be completed for all students in less than 40 min. It is thus ideally suited for a first time efficient yet psychometrically high-quality step in multiple-gating assessment. In a second gate, the longer ITRF could then be used for a more detailed clarification of the problems. Compared to the original ITRF, the teacher nomination step could, thus, be replaced by the systematic short screening developed here. Given this lower effort, the shortened version of the ITRF is more likely to be used in schools within multiple-gating procedures. Therefore, it contributes to the implementation of the regular assessment of children’s individual social and emotional development, as well as their specific needs.

The current study showed that reducing items and shortening questionnaires is applicable without sacrificing psychometric rigor. Previous studies from different fields have given similar examples on how a questionnaire can be reduced (Anthony et al. 2016; Becker et al. 2007; Chiesi et al. 2018; Volpe et al. 2011; Volpe and Gadow 2010). Based on these experiences, researchers developing questionnaires might always consider test efficiency and—if possible—prepare a short version for screening purposes in general. 

The present re-analysis is a further step in the development of a well-implementable, school-based behavioral screening. The items identified here for the short version need to be investigated in future studies with a different sample with regard to their factorial validity, their external evidence (especially convergent and divergent validity in comparison with other established scales), and their predictive power for the identification of actual behavioral problems. This seems particularly relevant in light of the fact that the extensive evidence on the construct validity of the longer ITRF has predominantly worked with the externalizing scales. A more in-depth analysis of the internalizing scales is yet to be conducted.

The results can be discussed against the background of teachers’ tendency to detect externalizing problems more easily than internalizing problems (Dwyer et al. 2006; Hartman et al. 2017). The focus of the test information shifted to a lower theta range (referring to less severe behavioral problems) stronger for internalizing than externalizing problems. Thus, the full versions of the externalizing scales, especially the OPD, were already strongly focused on a lower theta range, whereas the full internalizing scales focused more on a higher theta range (referring to students with severe internalizing problems). Selecting the items most sensitive for slightly above-average behavioral problems within the theta range of 0 to 1 affected the internalizing scales stronger than the externalizing scales. Moreover, the mean beta parameters of the internalizing items were higher than of the externalizing items. Lower beta parameters in the externalizing scales indicate that these items are more likely to be scored higher by teachers even if the behavioral problems are less severe. Conversely, higher beta parameters in the internalizing scales indicate that students need to have more severe internalizing behavioral problems for teachers to score the corresponding items higher. Thus, the results corroborate findings stating that teachers can detect externalizing problems better than internalizing problems.

### Limitations

The findings of the current study should be interpreted in the context of at least four limitations. First, the item reduction was merely based on the GPCMs and the parameters for discrimination and location on the theta range. This procedure pays little respect to the content of the items. For example, including an expert rating regarding the most relevant items of the original version of the ITRF would provide a broader empirical basis for the item selection.

Second, the revalidation of the shortened version did not examine external validity with other measures (e.g., other questionnaires assessing social, emotional, and behavioral problems). Investigating the external validity of the shortened version of the ITRF would improve the interpretability of the results.

Third, predictive validity was not investigated. As the shortened version of the ITRF is supposed to serve as a screener for social, emotional, and behavioral problems, its predicative validity is of great interest. Information on the accuracy with which the shortened version of the ITRF can predict social, emotional, and behavioral problems with different severity would increase the interpretability of the instrument. Moreover, this information might convince more teachers to implement an early assessment of risk for social, emotional, or behavioral problems.

Fourth, in our resulting models, items showed considerable local dependencies (LD). Even if this is a common problem in individual teacher ratings (Anthony et al. 2016; Wu 2017) and LD compromises the results of a GPCM only to a small degree (Song 2019), the results might be caused by specific rater effects, such as general tendencies or halo effects (Wu 2017). A potential solution might be psychometric evaluation approaches that allow to consider rater effects in behavior rating scales such as the many-facet Rasch model (see Anthony et al. 2022) or generalizability theory (e.g., Briesch et al. 2014). However, those approaches attempt quite strict a priori design specifications, which were not applied in the current study.

## 5. Conclusions

The results of the present study indicate that the assessment of students’ social, emotional, and behavioral risk is possible even with only a few items in the teacher rating. The scale used here is thus very well suited for the time-efficient measurement of students’ classroom behavior (90 s). This enables teachers to integrate behavioral diagnostics into their daily school routine and to identify students’ needs at an early stage in order to implement appropriate support services and prevent the development of psychosocial disorders. With the shortened version of the ITRF, applying early assessment of social, emotional, and behavioral development is facilitated in schools. 

## Figures and Tables

**Table 1:** Item parameters of the GPCMs for each subscale of the ITRF.

Item	α	β	τ_1_	τ_2_	τ_3_
Subscale AD					
I_2	1.665	1.669	−.648	.019	.629
I_7	1.745	1.522	−.692	.108	.584
I_8	1.665	1.623	−.553	.045	.509
**I_9**	1.774	1.468	−.773	.132	.641
I_10	1.282	1.859	−.080	−.082	.162
I_11	1.167	1.809	−.364	.117	.247
I_12	1.263	1.766	−.594	.077	.517
**I_15**	1.894	1.591	−.606	−.005	.611
I_17	1.345	1.789	−.613	.050	.562
I_19	1.848	1.833	−.333	.090	.243
**I_23**	2.357	1.549	−.647	.157	.490
Subscale SW					
I_1	2.635	1.543	−.657	.064	.593
**I_4**	4.289	1.568	−.757	.096	.661
**I_5**	3.998	1.671	−.662	.162	.500
**I_6**	3.440	1.929	−.836	.094	.741
I_13	1.198	1.807	−.789	.063	.593
I_14	1.155	1.843	−.477	.112	.365
I_16	1.679	1.526	−.638	.019	.619
I_24	1.323	1.822	−.644	.189	.455
Subscale OPD					
E_7	3.263	1.339	−.390	.043	.348
E_8	2.365	1.143	−.305	−.009	.314
**E_9**	2.136	.754	−.764	.098	.666
E_10	3.060	1.261	−.406	.026	.380
**E_11**	2.972	.823	−.715	.090	.625
E_12	1.879	.1292	−.491	−.035	.526
**E_13**	3.608	1.133	−.500	.059	.441
E_16	1.983	1.459	−.461	−.073	.534
Subscale APP					
**E_1**	2.007	.632	−.642	.047	.595
**E_2**	2.203	.866	−.613	.067	.546
E_3	2.518	1.062	−.522	.023	.498
**E_4**	3.034	1.054	−.693	.061	.632
E_5	2.167	.654	−.844	.258	.685
E_6	1.849	1.198	−.477	−.029	.505
E_14	2.576	1.351	−.519	.045	.475
E_15	.920	1.174	−.649	.075	.573

Note. AD = anxious/depressed behavior; SW = social withdrawal; OPD = oppositional/defiant problems; APP = academic productivity problems; bold items were selected for the shortened version.

**Table 2:** Item factor loadings and reliability of the shortened ITRF.

Item	α	β
**Anxious/Depressive**	**.73**	
Appears unhappy or sad		.76
Complains or whines		.65
Spends a lot of time worrying		.65
**Social Withdrawal**	**.87**	
Avoids social interactions		.86
Prefers to play alone		.84
Does not respond to others’ attempts to socialize		.80
**Oppositional/Defiant Behavior**	**.85**	
Disrupts others		.83
Has conflicts with peers		.81
Makes irrelevant comments		.80
**Academic Productivity Problems**	**.86**	
Does not complete classwork on time		.84
Does not start assignments independently		.91
Does not turn in class assignments		.74
**Model Fit** χ^2^ = 739.748, df = 48, *p* = .000; CFI = .954, TLI = .937, RMSEA = .075, C.I.RSMEA = [.70–.80]		

Note. α = Cronbach’s alpha; β = standardized factor loadings; df = degrees of freedom; CFI = comparative fit index; TLI = Tucker–Lewis index; RMSEA = root mean square error of approximation; CI = confidence interval.

**Table 3:** Factor correlations of the full and the short ITRF.

	Short ITRF				Full ITRF			
	AD	SW	ODP	APP	AD	SW	ODP	APP
**Short ITRF**								
AD		.56	.43	.41	.91	.59	.36	.34
SW			.18	.34	.47	.78	.16	.32
ODP				.52	.33	.27	.96	.50
APP					.34	.54	.42	.94
**Full ITRF**								
AD						.69	.37	.38
SW							.26	.52
ODP								.51

Note. AD = anxious/depressed behavior; SW = social withdrawal; OPD = oppositional/defiant problems; APP = academic productivity problems; all correlations were significant (*p* < .001).

## Data Availability

The data presented in this study are available on request from the corresponding author.

## Appendix A. Items of the ITRF (Bold Items Were Selected for the Shortened Version)

**E-1. Does not complete classwork on time (APD 1)**

**E-2. Does not start assignments independently (APD 2)**

E-3. Missing or incomplete homework (APD 3)

**E-4. Does not turn in class assignments (APD 4)**

E-5. Does not correct own work (APD 5)

E-6. Fails to pack needed materials for home (APD 6)

E-7. Argues with teacher (OPP 1)

E-8. Loses temper (OPP 2)

**E-9. Disrupts others (OPP 3)**

E-10. Uses inappropriate language (OPP 4)

**E-11. Has conflicts with peers (OPP 5)**

E-12. Bossy (OPP 6)

**E-13. Makes irrelevant comments (OPP 7)**

E-14. Comes to class unprepared (APD 7)

E-15. Does not participate in class (APD 8)

E-16. Does not respect others space (OPP 8)

I-1. Spends too much time alone (SW)

I-2. Complains about being sick or hurt (AD)

**I-4. Avoids social interactions (SW)**

**I-5. Prefers to play alone (SW)**

**I-6. Does not respond to others’ attempts to socialize (SW)**

I-7. Worries about unimportant details (AD)

I-8. Complains of headaches or stomach aches (AD)

**I-9. Appears unhappy or sad (AD)**

I-10. Clings to adults (AD)

I-11. Acts nervous (AD)

I-12. Acts fearful (AD)

I-13. Does not stick up for self (SW)

I-14. Overly shy (SW)

**I-15. Complains or whines (AD)**

I-16. Does not participate in group activities (SW)

I-17. Makes self-depreciating comments (AD)

I-19. Cries or is weepy (AD)

**I-23. Spends a lot of time worrying (AD)**

I-24. Slow to warm up to new people (SW)

## References

[B1-jintelligence-11-00057] Achenbach T. M., Becker Andreas, Döpfner Manfred, Heiervang Einar, Roessner Veit, Steinhausen Hans-Christoph, Rothenberger Aribert (2008). Multicultural assessment of child and adolescent psychopathology with ASEBA and SDQ instruments: Research findings, applications, and future directions. Journal of Child Psychology and Psychiatry.

[B2-jintelligence-11-00057] Achenbach T. M., McConaughy Stephanie H., Ivanova Masha Y., Rescorla Leslie A. (2011). Manual for the ASEBA Brief Problem Monitor.

[B3-jintelligence-11-00057] Anthony Christopher J., Di Perna James C., Lei Pui-Wa (2016). Maximizing Measurement Efficiency of Behavior Rating Scales Using Item Response Theory: An Example with the Social Skills Improvement System—Teacher Rating Scale. Journal of School Psychology.

[B4-jintelligence-11-00057] Anthony Christopher J., Styck Kara M., Volpe Robert J., Robert Christopher R. (2022). Using many-facet rasch measurement and generalizability theory to explore rater effects for direct behavior rating–multi-item scales. School Psychology online first.

[B5-jintelligence-11-00057] Aviles Ann M., Anderson Tanya R., Davila Erica R. (2006). Child and Adolescent Social-Emotional Development Within the Context of School. Child and Adolescent Mental Health.

[B6-jintelligence-11-00057] Becker Janine, Schwartz Carolyn, Saris-Baglama Renee N., Kosinski Mark, Bjorner Jakob Bue (2007). Using Item Response Theory (IRT) For Developing and Evaluating the Pain Impact Questionnaire (PIQ-6™). Pain Medicine.

[B7-jintelligence-11-00057] Berg Juliette, Nolan Elizabeth, Yoder Nick, Osher David, Mart Amy (2019). Social-Emotional Competencies in Context: Using Social-Emotional Learning Frameworks to Build Educators’ Understanding. Measuring SEL.

[B8-jintelligence-11-00057] Breitenstein Susan M., Hill Carri, Gross Deborah (2009). Understanding disruptive behavior problems in preschool children. Journal of Pediatric Nursing.

[B9-jintelligence-11-00057] Briesch Amy M., Swaminathan Hariharan, Welsh Megan, Chafouleas Sandra M. (2014). Generalizability theory: A practical guide to study design, implementation, and interpretation. Journal of School Psychology.

[B10-jintelligence-11-00057] Briesch Amy M., Ferguson Tyler David, Daniels Brian, Volpe Robert J., Feinberg Adam B. (2017). Examining the Influence of Interval Length on the Dependability of Observational Estimates. School Psychology Review.

[B11-jintelligence-11-00057] Bruhn Allison Leigh, Woods-Groves Suzanne, Huddle Sally (2014). A Preliminary Investigation of Emotional and Behavioral Screening Practices in K–12 Schools. Education and Treatment of Children.

[B12-jintelligence-11-00057] Burns John R., Rapee Ronald M. (2019). School-Based Assessment of Mental Health Risk in Children: The Preliminary Development of the Child RADAR. Child and Adolescent Mental Health.

[B13-jintelligence-11-00057] Casale Gino, Volpe Robert J., Daniels Brian, Hennemann Thomas, Briesch Amy M., Grosche Michael (2018). Measurement Invariance of a Universal Behavioral Screener Across Samples from the USA and Germany. European Journal of Psychological Assessment.

[B14-jintelligence-11-00057] Casale Gino, Volpe Robert J., Hennemann Thomas, Briesch Amy M., Daniels Brian, Grosche Michael (2019). Konstruktvalidität Eines Universellen Screenings Zur Unterrichtsnahen Und Ökonomischen Diagnostik Herausfordernden Verhaltens Von Schüler_innen–Eine Multitrait-Multimethod-Analyse. [Construct Validity of a Universal Screener to Economically Assess Students’ Behavior in the Classroom—A Multitrait-Multimethod-Analysis]. Zeitschrift für Pädagogische Psychologie [German Journal of Educational Psychology].

[B15-jintelligence-11-00057] Chiesi Francesca, Morsanyi Kinga, Donati Maria Anna, Primi Caterina (2018). Applying Item Response Theory to Develop a Shortened Version of the Need for Cognition Scale. Advances in Cognitive Psychology.

[B16-jintelligence-11-00057] Christensen Karl Bang, Makransky Guido, Horton Mike (2017). Critical Values for Yen’s Q3: Identification of Local Dependence in the Rasch Model Using Residual Correlations. Applied Psychological Measurement.

[B17-jintelligence-11-00057] Costello E. Jane (2016). Early detection and prevention of mental health problems: Developmental epidemiology and systems of support. Journal of Clinical Child & Adolescent Psychology.

[B18-jintelligence-11-00057] Daniels Brian, Volpe Robert J., Briesch Amy M., Fabiano Gregory A. (2014). Development of a Problem-Focused Behavioral Screener Linked to Evidence-Based Intervention. School Psychology Quarterly.

[B19-jintelligence-11-00057] Dineen Jennifer N., Chafouleas Sandra M., Briesch Amy M., McCoach D. Betsy, Newton Sarah D., Cintron Dakota W. (2022). Exploring Social, Emotional, and Behavioral Screening Approaches in U.S. Public School Districts. American Educational Research Journal.

[B20-jintelligence-11-00057] Domitrovich Celene E., Durlak Joseph A., Staley Katharine C., Weissberg Roger P. (2017). Social-Emotional Competence: An Essential Factor for Promoting Positive Adjustment and Reducing Risk in School Children. Child Development.

[B21-jintelligence-11-00057] Durlak Joseph A., Weissberg Roger P., Dymnicki Allison B., Taylor Rebecca D., Schellinger Kriston B. (2011). The Impact of Enhancing Students’ Social and Emotional Learning: A Meta-Analysis of School-Based Universal Interventions. Child Development.

[B22-jintelligence-11-00057] Dwyer Sarah B., Nicholson Jan M., Battistutta Diana (2006). Parent and teacher identification of children at risk of developing internalizing or externalizing mental health problems: A comparison of screening methods. Prevention Science.

[B23-jintelligence-11-00057] Eklund Katie, Renshaw Tyler L., Dowdy Erin, Jimerson Shane R., Hart Shelley R., Jones Camille N., Earhart James (2009). Early Identification of Behavioral and Emotional Problems in Youth: Universal Screening Versus Teacher-Referral Identification. California School Psychologist.

[B24-jintelligence-11-00057] Forness Steven R., Kim Joanne, Walker Hill M. (2012). Prevalence of Students with EBD: Impact on General Education. Beyond Behavior.

[B25-jintelligence-11-00057] Glover Todd A., Albers Craig A. (2007). Considerations for Evaluating Universal Screening Assessments. Journal of School Psychology.

[B26-jintelligence-11-00057] Goodman R. (1997). The Strengths and Difficulties Questionnaire: A Research Note. Journal of Child Psychology and Psychiatry.

[B27-jintelligence-11-00057] Halle Tamara G., Darling-Churchill Kristen E. (2016). Review of Measures of Social and Emotional Development. Journal of Applied Developmental Psychology.

[B28-jintelligence-11-00057] Hambleton Ronald K. (2000). Emergence of Item Response Modeling in Instrument Development and Data Analysis. Medical Care.

[B29-jintelligence-11-00057] Hartman Kelsey, Gresham Frank M., Byrd Shelby (2017). Student internalizing and externalizing behavior screeners: Evidence for reliability, validity, and usability in elementary schools. Behavioral Disorders.

[B30-jintelligence-11-00057] Kauffman James M. (1999). How We Prevent the Prevention of Emotional and Behavioral Disorders. Exceptional Children.

[B31-jintelligence-11-00057] Kendziora Kimberly T., Rutherford Robert B., Quinn Mary M., Mathur Sarup R. (2004). Early Intervention for Emotional and Behavioral Disorders. Handbook of Research in Emotional and Behavioral Disorders.

[B32-jintelligence-11-00057] Kilgus Stephen P., von der Embse Nathaniel P., Taylor Crystal N., Van Wie Michael P., Sims Wesley A. (2018). Diagnostic accuracy of a universal screening multiple gating procedure: A replication study. School Psychology Quarterly.

[B33-jintelligence-11-00057] Korpershoek Hanke, Harms Truus, de Boer Hester, van Kuijk Mechteld, Doolaard Simone (2016). A Meta-Analysis of the Effects of Classroom Management Strategies and Classroom Management Programs on Students’ Academic, Behavioral, Emotional, and Motivational Outcomes. Review of Educational Research.

[B34-jintelligence-11-00057] Kovess-Masfety Viviane, Husky Mathilde M., Keyes Katherine, Hamilton Ava, Pez Ondine, Bitfoi Adina, Carta Mauro Giovanni, Goelitz Dietmar, Kuijpers Rowella, Otten Roy (2016). Comparing the Prevalence of Mental Health Problems in Children 6–11 Across Europe. Social Psychiatry and Psychiatric Epidemiology.

[B35-jintelligence-11-00057] Lane Kathleen, Oakes Wendy Peia, Menzies Holly Mariah, Germer Kathryn A., Walker Hill, Gresham Frank M. (2014). Screening and identification approaches for detecting students at risk. Handbook of Evidence-Based Practices for Emotional and Behavioral Disorders: Applications in Schools.

[B36-jintelligence-11-00057] Langer David A., Wood Jeffrey J., Wood Patricia A., Garland Ann F., Landsverk John, Hough Richard L. (2015). Mental health service use in schools and non-school-based outpatient settings: Comparing predictors of service use. School Mental Health.

[B37-jintelligence-11-00057] Moore Stephanie A., Dowdy Erin, Hinton Tameisha, DiStefano Christine, Greer Fred W. (2022). Moving Toward Implementation of Universal Mental Health Screening by Examining Attitudes Toward School-Based Practices. Behavioral Disorders.

[B38-jintelligence-11-00057] Muraki Eiji, Linden Wim J., Hambleton Ronald K. (1997). A Generalized Partial Credit Model. Handbook of Modern Item Response Theory.

[B39-jintelligence-11-00057] Petermann Ulrike, Petermann Franz (2013). Lehrereinschätzliste für Sozial- und Lernverhalten.

[B40-jintelligence-11-00057] Polanczyk Guilherme V., Salum Giovanni A., Sugaya Luisa S., Caye Arthur, Rohde Luis A. (2015). Annual Research Review: A Meta-Analysis of the Worldwide Prevalence of Mental Disorders in Children and Adolescents. Journal of Child Psychology and Psychiatry, and Allied Disciplines.

[B41-jintelligence-11-00057] R Core Team (2022). R: A Language and Environment for Statistical Computing.

[B42-jintelligence-11-00057] Revelle William (2022). psych: Procedures for Personality and Psychological Research.

[B43-jintelligence-11-00057] Robitzsch Alexander, Kiefer Thomas, Wu Margaret (2022). TAM: Test Analysis Modules. https://CRAN.R-project.org/package=TAM.

[B44-jintelligence-11-00057] Sklad Marcin, Diekstra René, de Ritter Monique, Ben Jehonathan, Gravesteijn Carolien (2012). Effectiveness of School-Based Universal Social, Emotional, and Behavioral Programs: Do They Enhance Students’ Development in the Area of Skill, Behavior, and Adjustment?. Psychology in the Schools.

[B45-jintelligence-11-00057] Song Yoon Ah (2019). A Comparative Study of IRT Models for Rater Effects and Double Scoring. Doctoral dissertation.

[B46-jintelligence-11-00057] Splett Joni W., Garzona Marlene, Gibson Nicole, Wojtalewicz Daniela, Raborn Anthony, Reinke Wendy M. (2019). Teacher Recognition, Concern, and Referral of Children’s Internalizing and Externalizing Behavior Problems. School Mental Health: A Multidisciplinary Research and Practice Journal.

[B47-jintelligence-11-00057] Stiffler Meghan C., Dever Bridget V., Stiffler Meghan C., Dever Bridget V. (2015). Multiple-gating and mental health screening. Mental Health Screening at School: Instrumentation, Implementation, and Critical Issues.

[B48-jintelligence-11-00057] Volpe Robert J., Briesch Amy M. (2018). Establishing evidence-based behavioral screening practices in US schools. School Psychology Review.

[B49-jintelligence-11-00057] Volpe Robert J., Fabiano Gregory A. (2013). Daily Behavior Report Cards: An Evidence-Based System of Assessment and Intervention.

[B50-jintelligence-11-00057] Volpe Robert J., Gadow Kenneth D. (2010). Creating abbreviated rating scales to monitor classroom inattention-overactivity, aggression, and peer conflict: Reliability, validity, and treatment sensitivity. School Psychology Review.

[B51-jintelligence-11-00057] Volpe Robert J., Briesch Amy M., Chafouleas Sandra M. (2010). Linking Screening for Emotional and Behavioral Problems to Problem-Solving Efforts: An Adaptive Model of Behavioral Assessment. Assessment for Effective Intervention.

[B52-jintelligence-11-00057] Volpe Robert J., Briesch Amy M., Gadow Kenneth D. (2011). The efficiency of behavior rating scales to assess disruptive classroom behavior: Applying generalizability theory to streamline assessment. Journal of School Psychology.

[B53-jintelligence-11-00057] Volpe Robert J., Casale Gino, Mohiyeddini Changiz, Grosche Michael, Hennemann Thomas, Briesch Amy M., Daniels Brian (2018). A Universal Behavioral Screener Linked to Personalized Classroom Interventions: Psychometric Characteristics in a Large Sample of German Schoolchildren. Journal of School Psychology.

[B54-jintelligence-11-00057] Volpe Robert J., Yeung Tat Shing, Casale Gino, Krull Johanna, Briesch Amy M., Hennemann Thomas (2020). Evaluation of a German Language School-Based Universal Screening for Student Social, Emotional, and Behavioral Risk. International Journal of School & Educational Psychology.

[B55-jintelligence-11-00057] Walker Hill M., Small Jason W., Severson Herbert H., Seeley John R., Feil Edward G., Kettler Ryan J., Glover Todd A., Albers Craig A., Feeney-Kettler Kelly A. (2014). Multiple-Gating Approaches in Universal Screening Within School and Community Settings. Universal Screening in Educational Settings: Evidence-Based Decision Making for Schools.

[B56-jintelligence-11-00057] Whitcomb Sara A., Merrell Kenneth W. (2013). Behavioral, Social, and Emotional Assessment of Children and Adolescents.

[B57-jintelligence-11-00057] Wilson Mark (2004). Constructing Measures: An Item Response Modeling Approach.

[B58-jintelligence-11-00057] Wood Brandon J., Ellis Faith (2022). Universal Mental Health Screening Practices in Midwestern Schools: A Window of Opportunity for School Psychologist Leadership and Role Expansion?. Contemporary School Psychology.

[B59-jintelligence-11-00057] Wu Margaret (2017). Some IRT-Based Analyses for Interpreting Rater Effects. Psychological Test and Assessment Modeling.

